# Gallstones increase the risk of nonalcoholic fatty liver: A case‐control study

**DOI:** 10.1002/hsr2.70068

**Published:** 2024-11-05

**Authors:** Abdolreza Sheibani, Hamid Reihani, Ahmad shoja, Mohammad M. Gharibvand, Mohammad G. Hanafi

**Affiliations:** ^1^ Department of Radiology, School of Medicine Ahvaz Jundishapur University of Medical Sciences Ahvaz Iran; ^2^ School of Medicine Shiraz University of Medical Sciences Shiraz Iran; ^3^ Department of Radiology, School of Medicine South Khorasan University of Medical Sciences, Imam Reza Hospital Birjand Iran

**Keywords:** diabetes mellitus, gallstones, nonalcoholic fatty liver disease, risk factors

## Abstract

**Background:**

Nonalcoholic fatty liver disease (NAFLD) and gallstones are generally seen together, and many of the risk factors for fatty liver and gallstones are common and similar. Therefore, this study aims to investigate the relationship between NAFLD and gallstones.

**Methods:**

This case‐control study was conducted in patients referred to Imam Khomeini and Golestan hospitals of Ahvaz University of Medical Sciences in 2023, whose ultrasound showed fatty liver. Patients who were diagnosed with NAFLD by ultrasound were considered as the case group, and patients who did not have diagnostic findings of fatty liver in ultrasound were considered as the control group. Finally, the information recorded in the checklists was statistically analyzed using SPSS version 26 (SPSS Inc.).

**Results:**

Three hundred patients were included in our study, 150 as cases and 150 as controls. There was no significant difference between the groups in terms of gender and age (gender *P*‐value: 0.817/age P‐value: 0.102). A statistically significant relationship was found between obesity, diabetes mellitus (DM), the presence of gallstones, and NAFLD (weight *p*‐value < 0.001/DM *p*‐value < 0.001/gallstones *P*‐value: 0.03). In addition, based on binary logistic regression analysis, the presence of gallstones increases the odds of NAFLD by 2.33 times (*P*‐value: 0.035). Furthermore, having DM and increasing each BMI unit increases the odds of NAFLD by 16 times and 30%, respectively (BMI *p*‐value < 0.001/DM *p*‐value < 0.001).

**Conclusion:**

Based on the results of our study, gallstones are an important risk factor for NAFLD. The possible mechanisms are the existence of common risk factors between gallstones and NAFLD and the reduction of motility and flow of bile in the bile ducts with the presence of gallstones.

## 1 INTRODUCTION

Gallstones are the most common gallbladder disease, the diagnostic accuracy of which is about 90% with ultrasound.^1^ Deposition of calcium, bilirubin, cholesterol, and proteins are the cause of gallstone formation.^2^ The pathogenesis of gallstones includes changes in the composition of bile substances, stasis, and infection.^3^ When substances in the bile reach their maximum solubility, gallstones develop. These chemicals supersaturate the bile in the gallbladder, causing it to crystallize into small particles eventually.^4^ The incidence of gallstones is higher in women. It is related to risk factors such as age, obesity, hyperlipidemia, childbirth, contraceptive use, alcohol and smoking, family history, metabolic syndrome, and inactivity.^5^, ^6^, ^7^, ^8^ Its prevalence is about 5%–8% in Asian countries^9^ and about 10‐15% in the United States of America and Europe, and about 75% of patients are asymptomatic.^10^ However, every year, about one to two percent of people with gallstones need surgery, and this leads to imposing a significant medical and economic burden on society and the individual.^11^

One of the most frequent causes of chronic increase of liver enzymes and liver disease in adults and children is nonalcoholic fatty liver disease (NAFLD).^12^ Accumulation of fat in hepatocytes without underlying factors such as alcohol consumption, chronic viral hepatitis, and other liver diseases causes NAFLD.^13^ Cellular and molecular oxidative stress and hepatic iron and/or lipid peroxidation, as well as changes in the extracellular matrix, energy homeostasis, immune system function, and cytokine injury, are the molecular pathways of the development of NAFLD.^14^ This disease is generally asymptomatic and is diagnosed after the accidental discovery of increased liver enzymes.^15^ The disease begins with hepatic steatosis, which can progress to steatohepatitis, fibrosis, cirrhosis, and even liver cancer.^16^, ^17^, ^18^ In studies, the prevalence of NAFLD is estimated at 15‐20%, of which 22% progress to cirrhosis.^16^

NAFLD and gallstones are generally seen together^19^, ^20^, ^21^, ^22^ and have common risk factors such as type 2 diabetes, obesity, hyperlipidemia, and inactivity.^23^ Lu et al. reported in a study that NAFLD leads to increased complications of gallstones and cholecystectomy.^24^ Studies have reported that gallstones have a clear association with metabolic syndrome in elderly people with NAFLD.^25^ Considering the high prevalence of these two diseases and their heavy economic and treatment burden, prevention of the risk factors that cause these two diseases, in addition to preventing the occurrence of chronic liver and cardiovascular diseases, leads to a reduction in the number of biliary system surgeries. This study focuses on the association between gallstones and NAFLD as there haven't been many studies done on the risk of gallstones in fatty liver patients.

## 2 MATERIALS AND METHODS

### 2.1 Study setting

This case‐control study was conducted on patients from the population of Ahvaz who were examined by sonography and referred between January 2022 and December 2023. Before the study began, written informed consent was obtained from each participant. This study was conducted by random sampling among people who referred to Golestan and Imam Khomeini hospitals in Ahvaz city. People who were diagnosed with NAFLD by ultrasound were considered as the case group, and people who did not have diagnostic findings of fatty liver in ultrasound and were matched with people with fatty liver in terms of age and sex were considered as the control group. The ultrasound of the patients was performed and interpreted by an attending radiologist.

### 2.2 Sample size calculation

The sample size was calculated using the “power two proportions” module. Utilizing data from a pilot study, we estimated the sample size and computed the control‐case proportion using type I error (1.5), power (0.8), and alpha (0.05). Our NAFLD patients had a gallstone prevalence of (19%), and the controls had a gallstone prevalence of 7.7% in the pilot study. 142 was the minimum sample size we needed for each group to detect whether the stated difference exists between the two proportions. Therefore, we decided to include 150 for each group.

### 2.3 Inclusion and exclusion criteria and matching groups

Exclusion criteria included history of viral hepatitis, alcohol consumption, history of cholecystectomy, age under 20 years, patient dissatisfaction to participate in the study, prior biliary surgery, other significant hepatic pathologies, pregnancy, lactation, or severe comorbidities that could confound study outcomes. Inclusion criteria in the case group included age over 20 years, confirmed diagnosis of nonalcoholic fatty liver disease with sonography, and signing informed consent to participate in the study. To control for potential confounding variables, group matching was employed, stratifying participants by age and gender. This method aimed to enhance comparability between the case and control groups. To assess the independent associations between weight, height, BMI, history of diseases, and nonalcoholic fatty liver disease, these variables were not matched in the study design. By including these factors as covariates in the analysis, we aimed to identify their potential contributions to disease risk while controlling for the effects of age and sex through group matching.

### 2.4 Data gathering

Demographic and clinical data were collected using a standardized checklist by AS. Information regarding age, sex, weight, height, BMI, and medical history of diabetes mellitus and hypertension was obtained from patient medical records.

### 2.5 Statistical analysis

The information recorded in the checklists was statistically analyzed using SPSS version 26 (SPSS Inc.). Kolmogrov–Smirnove test was used to analyze the assumptions for the normality of quantitative data (When dealing with smaller sample sizes (less than 50), the Shapiro‐Wilk test is a more appropriate approach. However, for larger sample sizes, the Kolmogorov–Smirnov test is used).^26^ Qualitative variables were calculated as frequency (percentage), and quantitative variables were calculated with median (IQR) or mean ± SD. The Pearson chi‐square test, Fisher's exact test, and Man witney were used for analyses. A logistic regression model was applied to assess the association between developing NAFLD and the above‐mentioned factors. In the bivariate analysis, variables with a *P*‐value of less than 0.1 were chosen to be part of the binary logistic regression analysis. The adjusted odds ratio (OR) and 95% confidence interval (CI) were estimated. A *p*‐value less than 0.05 was considered statistically significant.

## 3 RESULTS

A total of 300 patients were collected as samples, of which 150 had NAFLD. These two groups were matched in terms of age and gender (gender P‐value: 0.817/age *P*‐value: 0.102). Among the people who had fatty liver (150 people), 78 people had grade 1 fatty liver (52%), 61 people had grade 2 fatty liver (40.7%), and 11 people had grade 3 fatty liver (7.3%). The median weight of the case group was 80 (19), and the control group was 68 (16), which showed that the control group was significantly heavier (*p*‐value < 0.001). 37 (24.7%) of NAFLD patients had diabetes, which was substantially more compared to 11 (7.3%) in healthy control group DM (*p*‐value < 0.001). 32 (21.3%) patients in the NAFLD group and 18 (12%) patients in the control group had gallstones, respectively, and this difference was statistically significant (*P*‐value: 0.03).

In summary, there was a statistically significant difference between the case and control groups in terms of weight, BMI, DM, and gallstones NAFLD (weight *p*‐value < 0.001/BMI *p*‐value < 0.001/DM *p*‐value < 0.001/gallstones *P*‐value: 0.03). All the information mentioned above is presented in Table 1.

**Table 1:** Comparison of demographic factors between case and control groups.

Variables		Control (*n* = 150)	Case (*n* = 150)	*P*‐value
		N (%) or median (IQR)	N (%) or median (IQR)	
Gender	Female	77 (51.3%)	79 (52.7%)	0.817
	Male	73 (48.7%)	71 (47.3%)	
Age (year)		52 (24)	48.5 (25)	0.102
Weight (kg)		68 (16)	80 (19)	<0.001
Height (cm)		164.5 (14)	167 (17)	0.118
BMI		24 (3)	28.8 (5.8)	<0.001
DM	Yes	11 (7.3%)	37 (24.7%)	<0.001
	No	139 (92.7%)	113 (75.3%)	
Htn	Yes	35 (23.3%)	49 (32.7%)	0.072
	No	115 (76.7%)	101 (67.3%)	
Gallstone	Yes	18 (12%)	32 (21.3%)	0.03
	No	132 (88%)	118 (78.7%)	

Abbreviations: BMI, Body mass index, DM, Diabetes mellitus; HTN, Hypertension; IQR, Interquartile range.

In the next step, variables with P‐values below 0.1 were entered into our binary logistic regression. Based on the analysis of the data obtained from this study, the odds ratio of the occurrence of nonalcoholic fatty liver with increasing BMI is equal to 1.5, which means that increasing the BMI of people leads to a 1.5‐fold increase in the occurrence of nonalcoholic fatty liver. Also, the odds ratio of nonalcoholic fatty liver disease in patients with diabetes is 11.07, which means that having diabetes leads to an 11‐fold increase in the incidence of nonalcoholic fatty liver disease. Our regression model had decent fitness regarding the omnibus test (*p*‐value < 0.001). Our Nagelkerke R Square was 0.531, indicating that the model is moderate to strong. Finally, the Hosmer and Lemeshow Test was also not significant (*P*‐value: 0.143).

**Figure 1:**
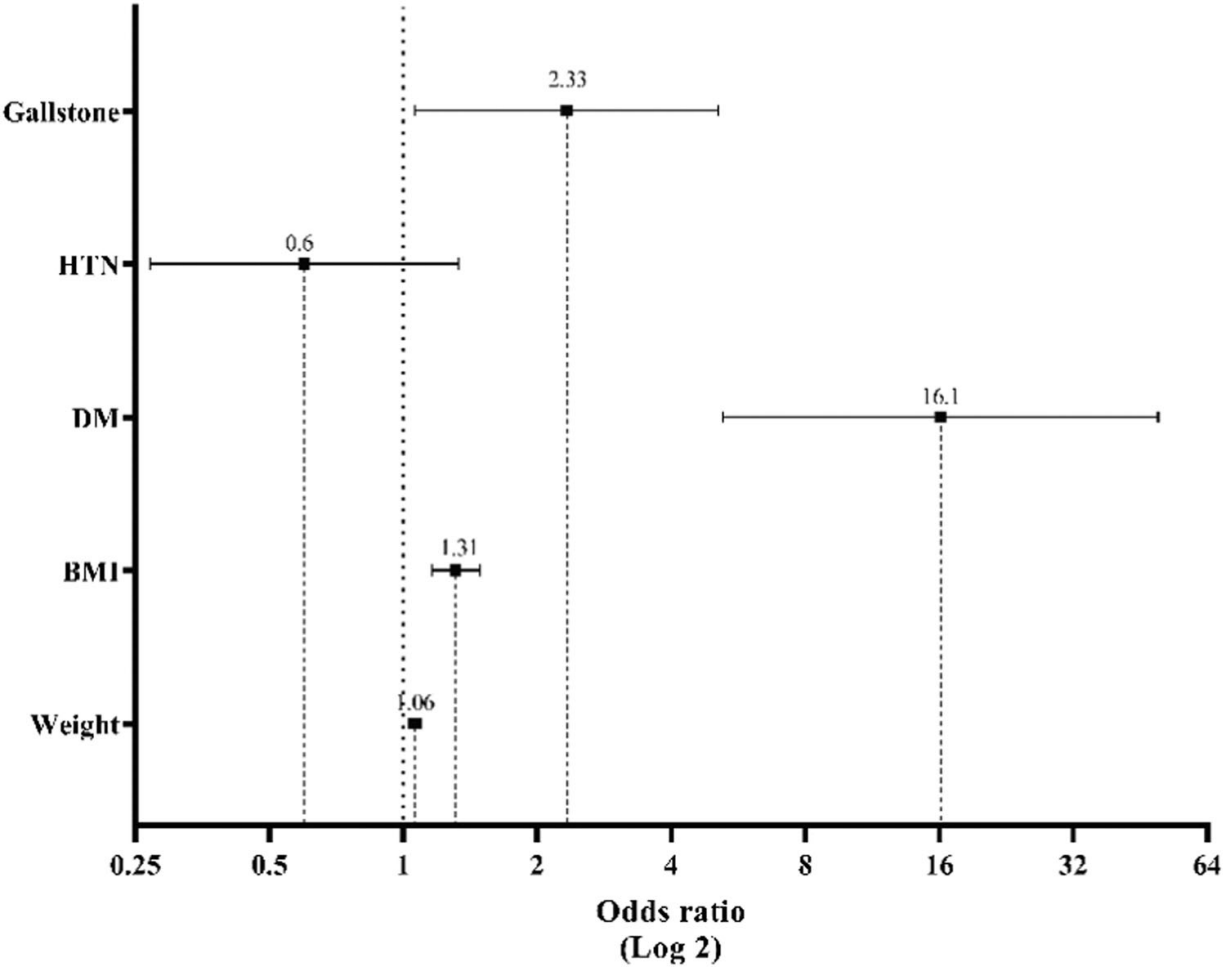
Forest plot figure of variables entered our regression.

According to logistic regression, each kilogram of weight gain can increase the odds of NAFLD by 6% (*p*‐value < 0.001). In addition, with each unit increase in BMI, the risk of NAFLD increases by 31% (*p*‐value < 0.001). According to our analysis, having HTN is not a significant risk factor for NAFLD (*P*‐value: 0.211). On the other hand, DM can increase the risk of NAFLD up to 16 times (*p*‐value < 0.001). And finally, patients diagnosed with gallstones on ultrasound have 2.33 times the chance of developing NAFLD (*P*‐value: 0.035). All these items are presented in Table 2. In addition, a forest plot figure is drawn to better visualize the effects (Figure 1).

**Table 2:** Examining the odds ratios of factors included in binary logistic regression analysis.

Variables	Regression coefficient	Standard error	Odds ratio	95% CI of OR	*P*‐value
Weight	0.066	0.019	1.06	1.03–1.10	<0.001
BMI	0.275	0.063	1.31	1.16–1.49	<0.001
DM	2.779	0.575	16.1	5.22–49.65	<0.001
HTN	−0.508	0.406	0.6	0.27–1.33	0.211
Gallstone	0.846	0.402	2.33	1.06–5.11	0.035

Abbreviations: BMI, Body mass index; CI, Confidence interval; DM, Diabetes mellitus; HTN, Hypertension.

## 4 DISCUSSION

According to the findings of this study, gallstones, obesity, and diabetes increase the risk of NAFLD. In various studies, the prevalence of obesity in patients with NAFLD has been reported between 30% and 100%, and the prevalence of type 2 diabetes has been reported between 10% and 75%.^13^, ^27^ In the present study, it was also shown that the risk of NAFLD in patients with higher BMI and diabetes is 1.31 times and 16 times, respectively. Moreover, the findings indicate a robust association between metabolic risk factors and NAFLD. Both overweight/obesity, as evidenced by elevated BMI and weight, and metabolic disturbances such as type 2 diabetes were independently associated with increased odds of NAFLD. These results underscore the importance of addressing metabolic health to mitigate the burden of NAFLD.

Based on studies, there is a two‐way relationship between fatty liver and gallstones. Gallstones are an independent risk factor for NAFLD. On the other hand, NAFLD itself is an independent risk factor for gallstones.^17^, ^28^, ^29^ The prevalence of gallstones in people with fatty liver in Italy, like the findings of the present study, was reported to be higher than the general population.^20^, ^30^ Chen et al. reported a statistically significant relationship between fatty liver and the incidence of gallstones.^31^ According to Sharifi et al.'s study, NAFLD and gallstones are clearly associated with each other. However, their findings showed no statistically significant difference based on gender in the prevalence of gallstones in NAFLD patients.^23^ The reason for the increased risk of NAFLD in patients with gallstones is two things: (a) these two conditions have very similar risk factors, and (b) gallstones cause a reduction in bile motility and flow, which can lead to a higher chance of fatty liver.^32^ It has also been shown that cholecystectomy can also increase the chance of fatty liver by the same mechanism.^33^ A study conducted among patients with a history of previous cholecystectomy reported an independent association with NAFLD, which was even stronger than the association with gallstones.^34^ In American studies, it has been shown that there is an independent relationship between NAFLD and cholecystectomy, which can be considered as a risk factor for NAFLD.^35^ In the study of Kichloo et al., it has been reported that there is a significant relationship between NAFLD and gallstones/cholecystectomy, and fatty liver is considered a risk factor for them.^36^ Finally, Patients with NAFLD had a higher frequency of gallstones, and those with patients with gallstones had a higher likelihood of developing NAFLD.^29^

Insulin resistance plays a central role in the occurrence of NAFLD.^17^ It should also be noted that the severity of insulin resistance affects liver histology in patients with NAFLD.^27^ According to Dharmalingam et al. study, the prevalence of NAFLD in diabetic patients is up to 70%.^37^ Studies have shown that diabetes increases the chance of NAFLD by increasing insulin resistance on the one hand and weight gain, which is common in diabetic patients on the other hand.^38^

In another study conducted by Sezgin et al.^39^ on 2797 participants in the Cappadocia cohort, the proportion of diabetic patients and hypertension was significantly higher in the group with hepatic steatosis. In our study, the frequency of diabetes and hypertension was higher in patients with NAFLD, but this relationship was statistically significant only for DM. Sezgin's study identified male gender, high blood pressure, and elevated BMI as risk factors for hepatic steatosis, while female gender, along with high blood pressure and elevated BMI, were identified as risk factors for gallstones. Our study was unable to examine gender and age as risk factors due to the matching of groups but aligned with Sezgin's findings regarding the association between increased BMI and NAFLD risk. Finally, both studies highlight the impact of metabolic factors, such as obesity and diabetes, as common risk factors for both conditions.^39^

### 4.1 Limitations

The small sample size was one of the limitations of this study. On the other hand, study participants could be matched with each other regarding more factors to minimize the difference between case and control and biases. Therefore, we suggest studies with a larger sample size with more matched variables. The causal relationship between gallstones and NAFLD is complex and not fully understood. It remains unclear whether one directly causes the other or not. for this purpose, stronger prospective cohort studies are needed.

## 5 CONCLUSION

Based on the results of this study, gallstones are an important risk factor for NAFLD. The possible mechanisms are the existence of common risk factors between gallstones and NAFLD and the reduction of motility and flow of bile in the bile ducts. In addition, DM and high BMI also play an essential role in increasing the risk of NAFLD.

## AUTHOR CONTRIBUTIONS

**Abdolreza Sheibani:** Conceptualization; methodology; data curation; investigation; funding acquisition; writing—original draft; writing—review and editing; validation. **Hamid Reihani:** Writing—original draft; writing—review and editing; conceptualization; methodology; formal analysis; data curation; investigation; validation; software; supervision. **Ahmad Shoja:** Writing—original draft; writing—review and editing; conceptualization; methodology; supervision; resources; project administration; validation. **Mohammad M. Gharibvand:** Writing—review and editing; writing—original draft; supervision; resources; validation; conceptualization; investigation. **Mohammad G. Hanafi:** Writing—original draft; writing—review and editing; project administration; resources; supervision; validation; investigation; conceptualization; methodology.

## CONFLICT OF INTEREST STATEMENT

The authors declare no conflict of interest.

## ETHICS STATEMENT

In our study, no intervention was performed on the patients. In addition, all patients were informed about the study procedures, and an informed consent form was obtained for the use of medical and ultrasound records. Moreover, the protocol of this study has been approved by the Ethics Committee of Ahvaz Jondishapur University of Medical Sciences by approval ID: IR. AJUMS. HGOLESTAN. REC.1402.166. An informed consent form was obtained from all patients.

## TRANSPARENCY STATEMENT

The lead author Mohammad Ghasem Hanafi affirms that this manuscript is an honest, accurate, and transparent account of the study being reported; that no important aspects of the study have been omitted; and that any discrepancies from the study as planned (and, if relevant, registered) have been explained.

## Data Availability

The datasets generated during the current study are available from the corresponding author upon reasonable request. All authors have read and approved the final version of the manuscript. Mohammad Ghasem Hanafi had full access to all of the data in this study and takes complete responsibility for the integrity of the data and the accuracy of the data analysis.
